# Microstructural spine alterations increase neuronal excitability in focal cortical dysplasia Type I

**DOI:** 10.3389/fncom.2026.1862383

**Published:** 2026-08-26

**Authors:** Jawon Gim, Na-Young Seo, Gyu Hyun Kim, Kea Joo Lee, Joon Ho Choi

**Affiliations:** 1 Neural Circuits Research Group, Korea Brain Research Institute (KBRI), Daegu, Republic of Korea; 2 Sensory and Motor System Research Group, Korea Brain Research Institute (KBRI), Daegu, Republic of Korea

**Keywords:** computational modeling, dendritic spine, epilepsy, NEURON, volume electron microscopy

## Abstract

**Introduction:**

Focal cortical dysplasia (FCD) is a leading cause of drug-resistant epilepsy and has predominantly been associated with impaired inhibitory signaling. However, recent ultrastructural studies have identified morphological alterations in excitatory synapses, suggesting that structural changes in excitatory connectivity may also contribute to cortical hyperexcitability.

**Methods:**

We used biophysically grounded computational models of human cortical pyramidal neurons to investigate how disease-associated alterations in dendritic spine architecture affect neuronal excitability. Spine density and spine geometry were independently manipulated based on quantitative volume electron microscopy measurements, allowing us to distinguish the contributions of individual structural features of excitatory synapses.

**Results:**

Reduced spine density and altered spine neck geometry increased neuronal excitability through complementary mechanisms, whereas variations in spine head size had comparatively minor effects. These structural alterations differentially influenced synaptic signal propagation and spike initiation. Their combined effects substantially increased neuronal output, particularly under conditions of sparse synaptic input.

**Discussion:**

These findings identify excitatory synaptic microstructure as an independent and mechanistically distinct contributor to hyperexcitability in FCD Type I. By linking ultrastructural abnormalities to altered neuronal input–output function, this study supports a potential contribution of excitatory synaptic alterations to the pathophysiology of FCD.

## 1 Introduction

Focal cortical dysplasia (FCD) is a severe malformation of neocortical development, and is one of the leading causes of drug-resistant epilepsy. Due to its pharmaco-resistant feature, surgical intervention is required for effective treatment (Riney et al., 2022; Chen et al., 2018; Kwan et al., 2011). FCD is characterized by abnormal cortical lamination and disorganized neuronal architecture, often accompanied by cytological abnormalities such as dysmorphic neurons and balloon cells (Cepeda et al., 2005). Such disruptions alter cortical circuit dynamics and establish FCD as a significant substrate for epileptogenesis (Cepeda et al., 2010; Coras et al., 2021; Kim and Choi, 2019). Electrophysiological recordings and immunohistochemical staining studies show disruption of the excitation-inhibition (E-I) balance within tissue from patients with FCD (Yang et al., 2021). Notably, a reduction in inhibitory signaling has been considered a principal contributor to epileptogenesis (Yang et al., 2021; Cho et al., 2024; Roper et al., 1999; Medici et al., 2016; Chen et al., 2025; Calcagnotto et al., 2005; Duma et al., 2024).

Focal cortical dysplasia Type I can cause severe drug-resistant epilepsy, yet its cellular mechanisms remain comparatively less well characterized (Yao et al., 2016). Its subtle histopathological alterations make this subtype particularly informative for determining whether changes in neuronal architecture alone can produce substantial functional hyperexcitability. Examining how alterations in excitatory neuronal structure affect the transformation of synaptic input into neuronal output may therefore provide insight into the mechanisms underlying epileptogenesis in FCD Type I. This question is especially relevant because epileptogenesis is a multiscale process spanning molecular, synaptic, circuit, and network scales (Eyal et al., 2018; Jean et al., 2023). At the microscopic level, molecular alterations including channelopathies and circuit remodeling can disrupt the balance between excitation and inhibition within neural networks (Lytton, 2008). At the macroscopic level, maladaptive dynamics in cortico-thalamic loops have been proposed to underlie seizure generation, with primary generalized seizures arising from nonlinear bistability that enables abrupt transitions from normal brain activity to large-scale synchronized oscillations (Breakspear et al., 2006). Despite differing views on the role of inhibitory mechanisms, studies converge on a well-known key scenario: an increase in the input–output firing rate relationship in FCD-affected cortical regions compared to normal cortex.

Adding further complexity, previous high-resolution electron microscopy (EM) studies of FCD Type I, a subtype lacking balloon cells, showed a reduction in excitatory synaptic density on pyramidal neurons (Kim et al., 2026; Rossini et al., 2023). In contrast to this overall decrease in synapse number, ultrastructural analyses also revealed a distinct population of enlarged excitatory synaptic contacts associated with extra-large spines. Relative to their counterparts in normal cortex, these synapses exhibited enlargement of both presynaptic and postsynaptic compartments, including a population of spines with head diameters greater than 1 μm and spine volumes exceeding 1 μm^3^. The number of synaptic vesicles in presynaptic compartments was 2.0-fold higher than that observed in normal cortex, suggesting an increased presynaptic capacity for neurotransmitter release. Moreover, the distribution of postsynaptic density (PSD) area was shifted toward larger values, consistent with a synaptic population biased toward stronger postsynaptic structural specialization. These opposing changes raise the question of whether reduced excitatory synapse density diminishes pyramidal neuron firing, or whether enlarged, structurally strengthened synapses compensate for this reduction and enhance the firing output of individual neurons.

Because a decrease in inhibitory synapse density doubtless increases neuronal firing rate, isolating the functional consequences of altered excitatory synapses experimentally is challenging. Most excitatory synapses in the cortex are formed onto dendritic spines. This pronounced geometric confinement plays a critical role in shaping synaptic signal integration (Harris and Kater, 1994). However, directly tracking voltage and ionic signal propagation within dendritic spines across different anatomical locations and as a function of spine morphology remains technically challenging due to their extremely small size and structural diversity (Tønnesen and Nägerl, 2016).

In this study, we use computational modeling to examine how signals propagate from dendritic spines into the parent dendrite, and how variations in spine morphology modulate this propagation by differentially shaping the contributions of individual anatomical compartments. Based on these simulations, we propose that individual features of spine architecture influence neuronal excitability through distinct mechanisms.

Our results further suggest that altered excitatory signal propagation may contribute to epileptogenic behavior under conditions in which inhibitory parameters are unchanged.

## 2 Methods

### 2.1 Common model backbone

Model cell morphology was based on previously described morphological features of human pyramidal neurons (Benavides-Piccione et al., 2024). This choice was motivated by the similarity of these features to neurons examined in electrophysiological (Cho et al., 2024) and volume electron microscopy studies (Kim et al., 2026) of FCD tissue.

**FIGURE 1:**
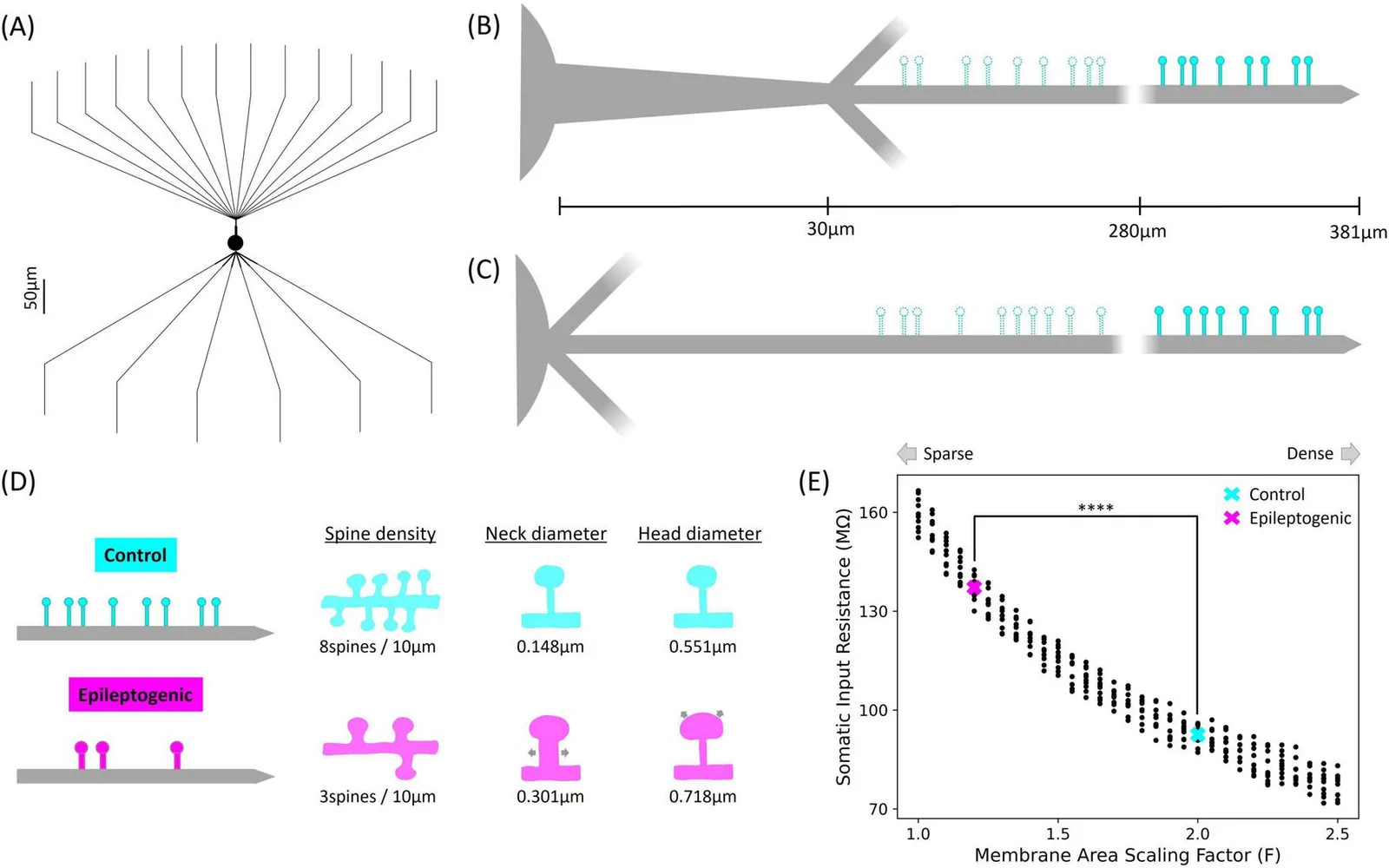
Model cell representation and somatic input resistance in the control and epileptogenic models. **(A)** Whole-cell morphology of the model neuron. **(B,C)** Apical **(B)** and basal **(C)** dendritic paths showing the spine-free, implicit spine (dashed), and explicit spine (solid) regions. A portion of each dendrite is omitted to visualize dendritic spines at an appropriate scale. Horizontal lines indicate distance from the soma. **(D)** Dendritic spine morphologies and densities in the control and epileptogenic models. **(E)** Somatic input resistance as a function of the membrane area scaling factor, F. Black dots indicate individual simulation runs, with 10 runs performed for each *F*-value. Cyan and magenta markers indicate the *F*-values corresponding to the representative control and epileptogenic morphologies, respectively. ****P < 0.0001.

We constructed two model cells representing neurons from histologically normal cortex and from seizure-focus cortex within a histologically confirmed dysplastic region, hereafter referred to as the control and epileptogenic models, respectively. Thus, the epileptogenic model was intended to represent cortical tissue that was both functionally identified as part of the seizure focus and histopathologically confirmed to contain focal cortical dysplasia. Both models shared the common model backbone described in this section, including the same morphological architecture, passive membrane properties, active somatic conductances, and spatial discretization. The models differed only in spine representation and the corresponding spine-related parameterization, as described in the following section. Briefly, the cell included a spherical soma with a diameter of 17 μm, 6 basal dendrites with a diameter of 1 μm and a length of 381 μm, and a single apical trunk 30 μm in length tapering into a tuft of 14 apical dendrites with a diameter of 1 μm and a length of 351 μm. Dendritic spines were distributed at distances greater than 30 μm from the soma (Figures 1A–C and Supplementary Table 1).

For implementation, each dendritic path was subdivided into three regions according to distance from the soma: a proximal 30 μm spine-free region, a middle 250 μm implicit spine region, and a distal 101 μm explicit spine region. In basal dendrites, these three regions partitioned the full 381 μm dendritic length. In the apical dendritic tree, the proximal 30 μm region corresponded to the apical trunk, whereas the 351 μm apical tuft dendrites were divided into the 250 μm implicit spine region and the 101 μm explicit spine region. The implementation of implicit and explicit spine representations is described in detail in the following section.

All dendritic compartments, including dendritic spines, were modeled with passive membrane properties. The soma additionally contained Hodgkin–Huxley–type sodium and potassium channels (Eyal et al., 2016). For all compartments, the axial resistance was set to 203.23 Ω⋅cm, the membrane capacitance to 1 μF/cm^2^, and the membrane resistance to 38,907 Ω⋅cm^2^. Somatic sodium and potassium conductances of the soma were set to 11,000 pS/μm^2^ and 2,100 pS/μm^2^, respectively (Supplementary Table 2). These values were initialized based on previously tested conductance ranges reported in studies of cortical pyramidal neurons (Eyal et al., 2016; Beaulieu-Laroche et al., 2018; Wilbers et al., 2023; Testa-Silva et al., 2022; Obi-Nagata et al., 2023) and subsequently refined to match the action potential kinetics measured in electrophysiological recordings from patient with FCD-derived samples (Cho et al., 2024).

To maintain numerical accuracy while improving computational efficiency, the number of segments (nseg) for each dendritic section was automatically determined based on the electrotonic length constant (λ) calculated from the passive membrane properties, following standard NEURON discretization principles (Hines and Carnevale, 2001). The soma was assigned a fixed nseg value of 3 (Supplementary Table 1).

### 2.2 Spine representation

Each dendritic path was divided into three regions: a proximal spine-free region, a middle implicit spine region, and a distal explicit spine region, as described above.

The proximal 30 μm of each dendritic path was defined as the spine-free region (Figures 1B,C). This design was based on previous morphological observations showing that dendritic spines are rare in proximal dendritic segments of human pyramidal neurons (Benavides-Piccione et al., 2024).

The distal 101 μm of each dendritic path was implemented as the explicit spine region, in which individual dendritic spines were modeled with full geometrical detail based on spine dimensions and density reported in a volume electron microscopy study of FCD tissue (Kim et al., 2026; Figures 1B,D and Supplementary Table 1). Spines were randomly distributed within the explicit spine region at model-specific densities of 8 and 3 spines per 10 μm for the control and epileptogenic models, respectively.

Because a substantial fraction of spines in the volume electron microscopy dataset exhibited noncanonical morphologies with poorly defined head–neck boundaries, spine neck length could not be reliably quantified as an independent parameter. To preserve the measured morphological characteristics while minimizing additional geometric assumptions, spine neck length was defined operationally as the difference between total spine length and spine head diameter.

In the control model, each dendritic spine consisted of a spherical head and a cylindrical neck, with a total spine length of 1.823 μm. The spine head had a diameter of 0.551 μm and was connected to a cylindrical neck with a diameter of 0.148 μm and a length of 1.272 μm. In the epileptogenic model, dendritic spines were modeled as larger and thicker but shorter than control spines, based on the same volume electron microscopy dataset. Each epileptogenic spine had a head diameter of 0.718 μm, a neck diameter of 0.301 μm, a neck length of 0.978 μm, and a total spine length of 1.696 μm. Each spine head was represented by one segment, whereas each spine neck was divided into three segments to resolve voltage attenuation between the spine head and the dendritic shaft.

The middle 250 μm of each dendritic path was implemented as the implicit spine region. This region was treated as a spiny dendritic segment but did not contain individually resolved spine geometry in order to reduce computational complexity. Instead, passive membrane properties in this region were adjusted using a surface-area scaling factor, F, to account for the additional membrane area contributed by dendritic spines (Eyal et al., 2018). The same model-specific spine densities used in the explicit spine region were also used to estimate the spine-related surface-area load in the implicit spine region. The factor F was defined as the ratio of the combined dendritic shaft and spine surface area to the dendritic shaft surface area:


$$F=\frac{\left(d\,e\,n\,d\,r\,i\,t\,i\,c\,s\,h\,a\,f\,t\,s\,u\,r\,f\,a\,c\,e\,a\,r\,e\,a+s\,p\,i\,n\,e\,s\,u\,r\,f\,a\,c\,e\,a\,r\,e\,a\right)}{d\,e\,n\,d\,r\,i\,t\,i\,c\,s\,u\,r\,f\,a\,c\,e\,a\,r\,e\,a}$$

For the control model, F was set to 2.0, consistent with the previously reported range of 1.78–2.39 estimated from confocal image analysis of human cortical pyramidal neurons (Eyal et al., 2016). For the epileptogenic model, F was set to 1.2, calculated from the spine geometry and density described above using standard geometrical surface-area equations (Supplementary Equations 1, 2).

Alpha-amino-3-hydroxy-5-methyl-4-isoxazolepropionic acid (AMPA) and N-methyl-D-aspartate (NMDA) receptors were assigned to the head of dendritic spines to deliver synaptic activation. Receptor conductance parameters were set based on values previously reported in related studies (Cho et al., 2024; Grunditz et al., 2008; Testa-Silva et al., 2022; Obi-Nagata et al., 2023).

Synaptic activation was restricted to spines within the explicit spine region, while the implicit spine region served to preserve the global electrical load imposed by spines without resolving individual spine geometry.

### 2.3 Simulation configurations

All simulations were based on the common model backbone described above, with configuration-specific modifications as described below.

#### 2.3.1 Single-spine activation protocol

All single-spine simulations were performed using the common model backbone and spine representations described above. A single excitatory synapse was activated on a dendritic spine with explicit geometry, and the resulting voltage responses were recorded at the spine head, spine base (dendritic shaft), and soma (Supplementary Figure 1A).

The location of the activated spine was systematically assigned at regular intervals across both the explicit and implicit spine regions, taking into account the electrotonic length constant (λ) determined by the passive membrane properties. Voltage responses from deployed locations were compiled into a single dataset and then resampled to match the target sample size. Resampling was performed in proportion to the apical-to-basal dendritic composition of the cell morphology, thereby avoiding under- or overrepresentation of their respective contributions. In all cases, the activated spine itself was represented with full anatomical detail.

To isolate the contribution of individual spine morphological features, single-spine simulations were performed under three controlled configurations, in which only one parameter was varied at a time while all others were held constant at control conditions. Synaptic conductance parameters for AMPA and NMDA receptors were fixed to control conditions across all single-spine simulations.

Each simulation condition was repeated across multiple independent runs. The number of simulations performed for each condition and the number of data points after resampling are indicated in the corresponding figure legends.

##### 2.3.1.1 Spine density variation

Spine density was varied by adjusting the factor F applied to all dendritic compartments located more than 30 μm from the soma, irrespective of whether they belonged to the explicit or implicit spine regions. This scaling was implemented to mimic global changes in spine density across the common model backbone. *F*-values ranged from 1.0 to 2.5.

The activated spine was placed independently of the factor F and was represented with explicit geometry. To examine the relationship between spine density and electrophysiological responses, simulations were performed across the full range of factor F. For statistical comparisons, simulations corresponding to control and epileptogenic models were repeated without duplication across multiple runs and summarized using violin plots.

##### 2.3.1.2 Spine neck diameter variation

Spine neck diameter was varied independently to examine its effects on voltage attenuation between the spine head and base. For statistical comparisons, simulations corresponding to control and epileptogenic models were repeated without duplication across multiple runs and summarized using violin plots.

##### 2.3.1.3 Spine head diameter variation

Spine head diameter was varied independently to examine its effects on EPSP amplitudes. Simulations corresponding to control and epileptogenic models were repeated for statistical comparison and summarized using violin plots.

#### 2.3.2 Multi-spine activation and input–output simulations

Multi-spine simulations were performed to examine how spine morphology influences neuronal firing and input–output relationships at the whole-cell level. All simulations used the common model backbone described above. In these simulations, dendritic spines with explicit geometry were placed exclusively within the explicit spine region. Spine morphology and synaptic conductance parameters differed between control and epileptogenic models, while all other cellular properties were held constant (Supplementary Tables 1, 2).

##### 2.3.2.1 Synchronous multi-spine activation

Synchronous multi-spine activation simulations were performed to estimate the firing threshold, defined as the minimum number of simultaneously activated spines required to elicit a somatic action potential. For each simulation run, spine placement and the subset of activated spines were randomized. The number of activated spines was systematically increased across simulations to determine firing threshold for each model.

Each simulation was repeated across multiple independent runs. The number of simulations performed for each condition is indicated in the corresponding figure legends.

##### 2.3.2.2 Input–output rate simulations

Input–output simulations were performed to characterize the relationship between synaptic input rate and somatic firing rate. For each simulation run, spine placement was randomized, and all spines received synaptic inputs generated as independent Poisson processes. Input rates were varied from 0.2 to 5.0 Hz.

Each simulation lasted 12 s, and somatic spikes occurring between 1 and 10 s were counted to compute firing rates. Somatic firing rate was quantified as a function of input rate to generate input–output curves for each model. Each simulation was repeated across multiple independent runs. The number of simulations performed for model is indicated in the corresponding figure legends.

### 2.4 Somatic input resistance measurement

Somatic input resistance was measured using the common model backbone described in Methods 2.1 and the spine representations described in Methods Section 2.2. Across simulations, the total number of dendrites was fixed at 20, and the basal-to-apical dendrite composition was varied across simulations.

Somatic input resistance was determined using a standard protocol (Stuart and Spruston, 1998) by injecting a 100 pA somatic current starting at 200 ms and lasting 500 ms. Baseline membrane potential was defined as the mean voltage during the 30 ms preceding current injection, and steady-state voltage as the mean voltage over the final 90 ms of the pulse, excluding the last 10 ms. Somatic input resistance was computed as the steady-state voltage deflection divided by the injected current.

### 2.5 Statistics

Statistical analyses were performed using GraphPad Prism. Two-sided unpaired *t*-tests were applied to comparisons of somatic input resistance (Figure 1E). All other comparisons were evaluated using two-sided Mann–Whitney U tests. Statistical significance was defined as *P* < 0.05, with **** indicating *P* < 0.0001.

**FIGURE 2:**
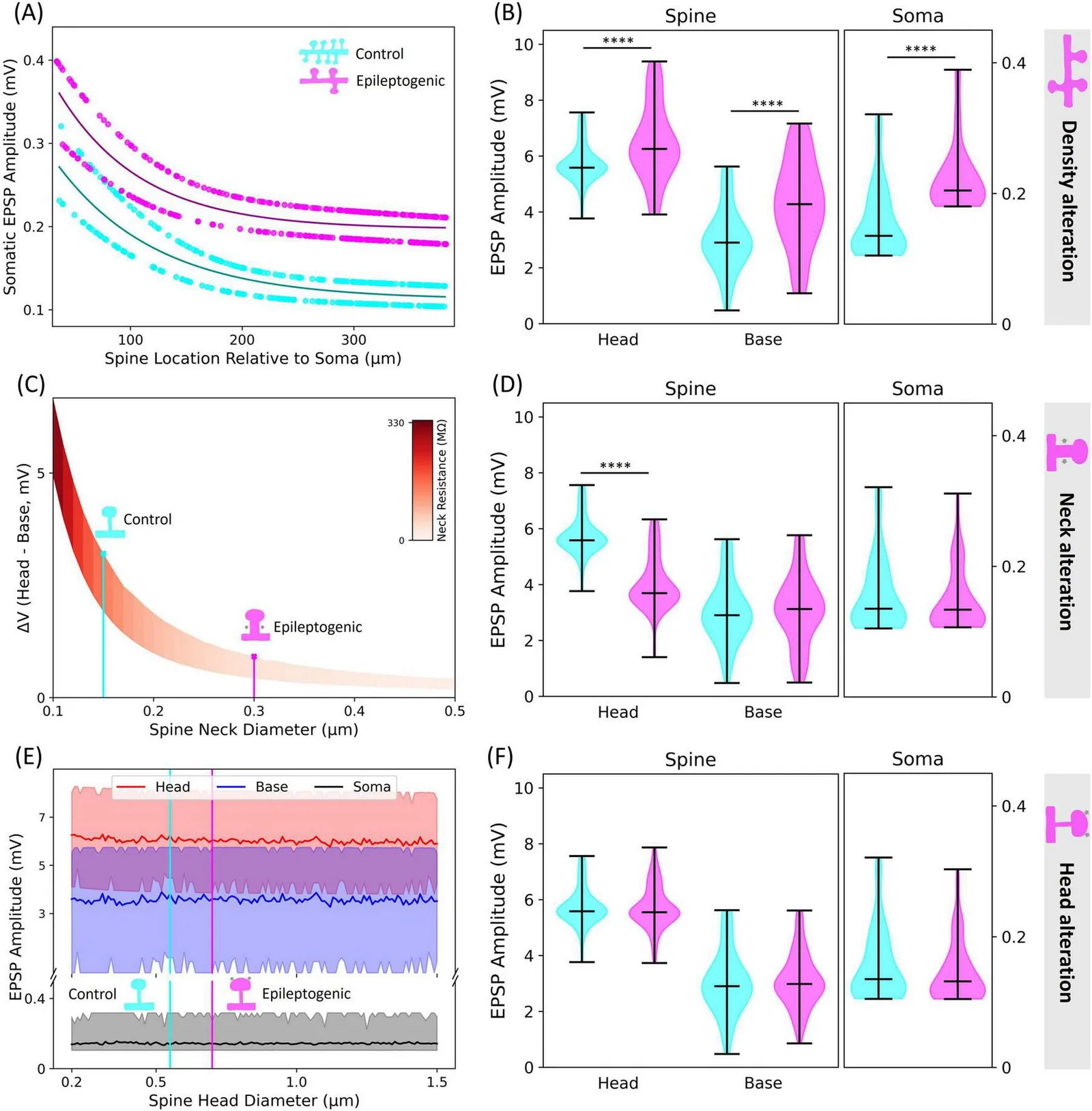
Cellular responses associated with model-specific differences in dendritic spine morphology and density. **(A)** Relationship between the distance from the soma to the activated spine and the resulting somatic EPSP amplitude. Dotted lines indicate individual simulation results, and solid lines indicate exponential fits to the data. For each model, the upper and lower dotted traces correspond to activation of basal and apical dendritic spines, respectively. A total of 300 simulations were performed for each model. **(C)** Voltage difference between the spine head and base as a function of spine neck diameter. Curves are color-coded by spine neck resistance (See Supplementary Equation 3). A total of 100 simulations were performed for each neck diameter value. **(E)** EPSP amplitude as a function of spine head diameter. A total of 100 simulations were performed for each head diameter value. In panels **(C,E)**, cyan and magenta markers indicate the model-specific spine morphologies corresponding to the control and epileptogenic models, respectively. **(B,D,F)** Violin plots summarizing EPSP amplitudes following single-spine activation in models differing in spine density **(B)**, spine neck diameter **(D)**, and spine head diameter **(F)**. EPSP amplitudes are shown at the spine head, spine base, and soma. Cyan and magenta indicate the control and epileptogenic models, respectively. For panel **(B)**, 80 and 60 simulations were performed for the control and epileptogenic models, respectively. For panels **(D,F)**, 80 simulations were performed for each model. For all violin plots, the central horizontal line indicates the median. ****P < 0.0001.

In Figures 2B,D,F, horizontal lines within the violin plots indicate the maximum, median, and minimum values from top to bottom, respectively.

### 2.6 Simulation

All simulations in this paper were conducted in NEURON (v8.2) (Hines and Carnevale, 2001) with Python (v3.10) (Python Software Foundation, 2021).

## 3 Results

### 3.1 Validation of model cell properties

To establish the validity of the model cell, we assessed its basic electrophysiological properties by measuring somatic input resistance (Figure 1E; Methods 2.4). Somatic input resistances in the control and epileptogenic models were 89.4 ± 0.93 and 130.45 ± 1.33 MΩ (mean ± SEM), respectively. Although the model did not reproduce the absolute input resistance values observed in corresponding human electrophysiological recordings by Cho et al. (2024) (109.4 ± 7.0 and 156.7 ± 8.7 MΩ for controls and patients with FCD, respectively), it preserved the relative differences between groups. Considering that the model cell differed from real neurons in morphological details such as branching pattern and did not include a full complement of ion channels, the comparable difference in somatic input resistance between the two models suggests that the model appropriately reflected the distinction between them.

### 3.2 Spine density–dependent modulation of somatic input resistance and EPSP amplitude

We next investigated how differences in spine density shape passive electrical properties and, in turn, modulate EPSP amplitude (Figures 1E, 2A,B), using the protocol described in Methods Section 2.3.1.1. In the model, spine density-associated changes were represented through the membrane area scaling factor, F (Methods Section 2.2). Although F is not equivalent to spine density alone, because it incorporates both spine density and spine geometry, this formulation captures the primary passive effect of spine loss in the absence of synaptic input: a reduction in dendritic membrane surface area. As shown in Figure 1E, the reduction in spine density, represented by a corresponding decrease in the membrane area scaling factor F, was associated with a systematic increase in somatic input resistance. This relationship reflects the passive membrane-area effect of spine loss: with passive channel density held constant, a reduction in membrane area decreases total leak conductance, thereby increasing input resistance.

Importantly, as mentioned in the previous section “3.1 Validation of model cell properties,” input resistance was significantly higher in the epileptogenic model (130.45 ± 1.33 MΩ) than in the control model (89.4 ± 0.93 MΩ; two-sided unpaired *t*-test, *P* < 0.0001). This effect was observed despite identical dendritic tree morphology and total dendritic length across models, indicating that differences in spine architecture alone are sufficient to produce substantial shifts in input resistance (Figure 1E). Because input resistance sets the gain with which synaptic inputs are translated into voltage responses, this scaling predicts model-dependent differences in EPSP amplitude even for identical synaptic input strength.

Consistent with this prediction, EPSPs evoked by the same synaptic activation were significantly larger in the epileptogenic model at the spine head (5.58 vs. 6.25 mV), spine base (2.90 vs. 4.27 mV), and soma (0.125 vs. 0.204 mV; all medians, control vs. epileptogenic; Mann-Whitney U-test, all *P* < 0.0001; Figure 2B, Supplementary Figures 1B,C). Somatic EPSP attenuation was also lower in the epileptogenic model than in the control model (Figure 2A).

Together, these results indicate that reduced spine density, represented through a lower membrane area scaling factor, passively increases input resistance by decreasing the effective dendritic membrane surface area. This, in turn, promotes greater preservation of excitatory signals in the epileptogenic model, enhancing the somatic impact of individual synaptic inputs.

### 3.3 Spine morphology–dependent regulation of electrical compartmentalization

We next examined how changes in spine morphology influence electrical compartmentalization between the spine head and the parent dendrite, using the simulation protocols described in Methods Sections 2.3.1.2–2.3.1.3. To quantify the degree of compartmentalization, we analyzed the voltage difference between the spine head and base (ΔV_*head*–*base*_) as a function of spine neck diameter (Figure 2C). Increasing neck diameter was associated with a progressive reduction in ΔV_*head*–*base*_, indicating weakened electrical isolation of the spine head from the dendritic shaft.

Consistent with this relationship, comparisons of EPSP amplitude across models revealed distinct spatial distribution of voltage. The control model exhibited significantly larger EPSP amplitudes in the spine head (5.58 vs. 3.69 mV; *P* < 0.0001), whereas EPSP amplitudes at the spine base remained effectively unchanged between models (2.90 vs. 3.12 mV; *P* = 0.3564; Figure 2D, Supplementary Figures 1B,D). This shift is explained by the reduction in axial resistance between the spine head and dendritic shaft as neck diameter increases, which facilitates current flow out of the spine head (Figure 2C, colormap). Accordingly, for spines of the same length, axial resistance was more than threefold higher in the control model than in the epileptogenic model.

Importantly, this effect was progressively attenuated along the spine-to-soma pathway: the difference was most pronounced at the spine head (*P* < 0.0001), minimal at the spine base (*P* = 0.3564), and not noticeable at the soma (0.135 vs. 0.133 mV; *P* = 0.6270). This spatial attenuation indicates that spine neck diameter predominantly modulates electrical compartmentalization at the spine head–dendritic shaft interface, with negligible influence on downstream dendrite-to-soma signal propagation.

In contrast, no significant differences in EPSP amplitude were observed at any measurement site after varying spine head size (head: *P* = 0.3745, base: *P* = 0.5361, soma: *P* = 0.2944; Figures 2E,F), indicating that head morphology enlargement alone is insufficient to alter voltage propagation under these conditions.

**FIGURE 3:**
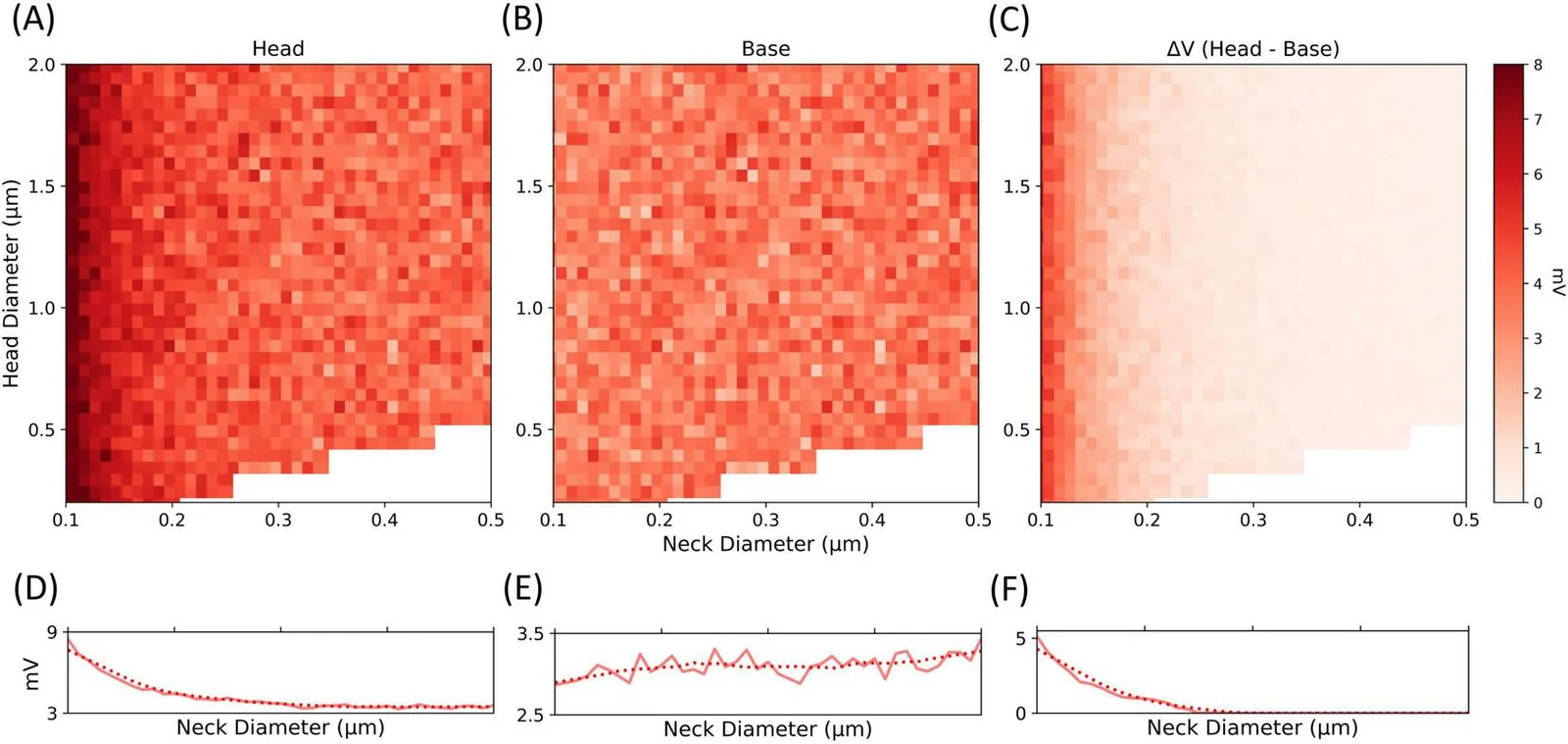
Spine neck diameter predominantly determines voltage compartmentalization across a broad range of spine geometries. **(A–C)** Heatmaps showing EPSP amplitudes as functions of spine neck diameter and spine head diameter. Panels show EPSP amplitude at the spine head **(A)**, EPSP amplitude at the spine base **(B)**, and the voltage difference between the spine head and base (ΔV_*head*–*base*_) **(C)**. **(D–F)** Corresponding line plots showing values averaged across spine head diameters as a function of spine neck diameter for spine head EPSP amplitude **(D)**, spine base EPSP amplitude **(E)**, and (ΔV_*head*–*base*_) **(F)**. Solid lines indicate mean values, and dashed lines indicate smoothed means. 30 independent simulations were performed for each neck–head diameter combination.

To further determine whether electrical compartmentalization is governed by absolute or relative spine geometry, we examined ΔV_*head*–*base*_ across a broad range of spine neck and head diameter combinations using the same simulation protocol (See Methods 3.2.1; Figure 3). This analysis showed that ΔV_*head*–*base*_ was determined primarily by the absolute value of spine neck diameter, with largely independence on spine head diameter across the range tested. In particular, contours of similar ΔV_*head*–*base*_ aligned predominantly along the neck-diameter axis rather than the head-diameter axis, with no evident diagonal trend, indicating that electrical coupling was set mainly by absolute neck diameter. Consistent with this interpretation, variations in the spine head-to-neck ratio did not produce a systematic effect on electrical compartmentalization. Thus, relative geometric scaling between head and neck was not a major determinant of voltage isolation.

As a complementary analysis, we further examined EPSP compartmentalization across paired variations in spine neck diameter and neck length (Supplementary Figures 2A–C). This analysis showed that the contribution of neck length was strongly constrained by neck diameter. When neck diameter was within or below the control-model range, increasing neck length increased head–base voltage separation, consistent with increased axial resistance along the spine neck. However, this length-dependent effect was progressively attenuated as neck diameter increased. This pattern was further supported by the calculated spine neck resistance landscape, in which axial resistance decreased steeply with increasing neck diameter and varied appreciably with neck length primarily in the narrow-diameter range (Supplementary Figure 2D). At neck diameters above approximately 0.2 μm, changes in neck length produced only modest changes in axial resistance and EPSP compartmentalization across the tested range.

Collectively, these results indicate that spine neck diameter is the dominant structural determinant of head–shaft voltage compartmentalization, because it strongly constrains spine neck axial resistance. Neck length contributes to voltage isolation in a diameter-dependent manner, with its effect being most evident in narrow-neck spines. In contrast, spine head size and relative head-to-neck scaling had comparatively limited effects under the tested conditions.

### 3.4 Spine-dependent modulation of spike threshold and input–output gain

**FIGURE 4:**
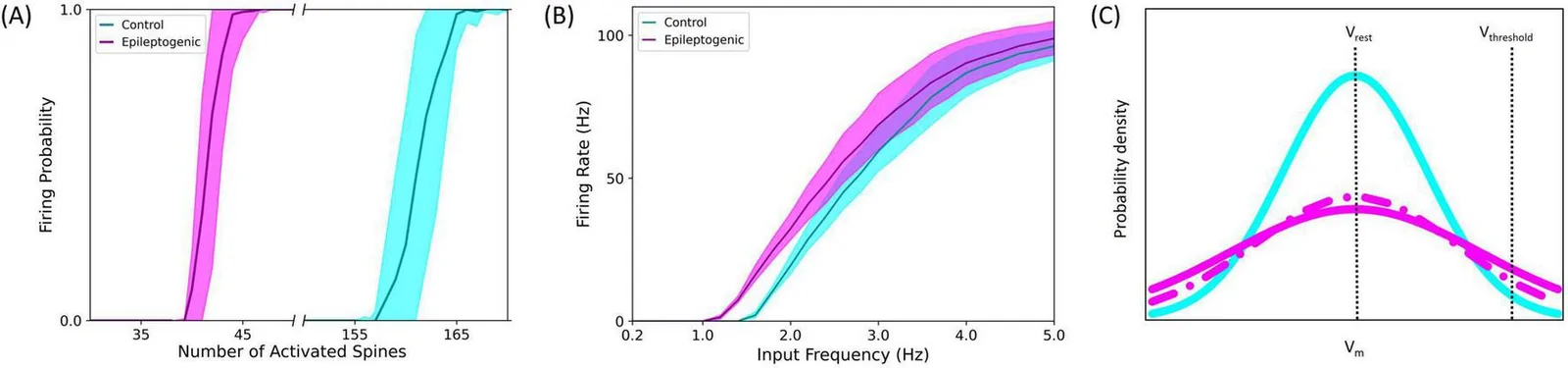
Spine-dependent modulation of neuronal excitability and input–output gain. **(A)** Firing probability as a function of the number of simultaneously stimulated spines. For each number of stimulated spines, 50 simulations were performed for each model. **(B)** Input–output firing rate curves in response to stochastic synaptic input delivered independently to explicit spines. Solid lines represent the mean somatic firing rate. For each input frequency, 30 simulations were performed for each model. **(C)** Schematic illustration of the membrane voltage distribution in the control and epileptogenic models. The probability density of somatic membrane voltage is shown as a function of membrane voltage, with the spike threshold indicated. Cyan solid line represents membrane potential distribution of control neuron. Magenta dashed line for FCD spine density and size effect and magenta solid line representing total effect on V_*m*_ distribution.

To determine how spine-level integration impacts spike generation, we quantified the action potential threshold during synchronous spine activation using the protocol described in Methods Section “2.3.2 Multi-spine activation and input–output simulations.” The control model required activation of 157 spines to elicit a spike, whereas the epileptogenic model reached spike initiation with only 41 spines (Figure 4A), corresponding to a 3.82-fold reduction in the number of activated spines required for spike initiation. When normalized to the total number of explicit spines, this corresponded to 9.8% activation in the control model and 6.8% in the epileptogenic model. These results indicate that spine-dependent changes substantially lower the effective spike threshold, increasing neuronal excitability at the level of convergent synaptic input.

We next examined how this lowered effective threshold reshapes the neuronal input–output relationship. Across the tested range of input frequencies (0.2–5 Hz), the epileptogenic model consistently exhibited higher mean firing rates than the control model (Figure 4B). Notably, this difference was input-regime dependent: at higher input rates (≥3.4 Hz), firing rates differed only modestly (1.04-fold), whereas under sparse input (<3.4 Hz), firing in the epileptogenic model was markedly enhanced, reaching up to 2.15-fold higher than control in average.

Together, these results show that spine-dependent alterations selectively amplify neuronal output in low-input regimes by reducing the effective spike threshold, while exerting relatively minor effects when synaptic drive is strong.

## 4 Discussion

In this study, we used biophysically grounded computational modeling to examine how alterations in dendritic spine architecture shape synaptic signal propagation and neuronal excitability. By explicitly incorporating spine morphological parameters directly derived from volume electron microscopy of human FCD Type I tissue (Kim et al., 2026), our approach avoids reliance on idealized or arbitrarily chosen geometric values and instead reflects disease-associated structural changes observed experimentally.

Based on these EM data, we hypothesized that morphological variability in excitatory synapses alone could contribute to epileptogenesis. To formalize this hypothesis, we considered a simplified stochastic model in which synaptic input arrives as a Poisson process. To render this assumption biologically interpretable, we consider the effective EPSP rate to scale with the total number of excitatory synapses, which in pyramidal neurons is well approximated by dendritic spine density. Under this interpretation, reductions in spine number observed in FCD Type I tissue would correspond to a proportional decrease in synaptic event rate. Conversely, we assume that the EPSP amplitude w scales with the strength of individual synapses, which is strongly influenced by spine head volume and associated postsynaptic density size. The presence of hypertrophic spines in FCD Type I therefore implies an increase in the effective synaptic size.

Under these biological assumptions-namely that ν ∝ total spine number and w ∝ spine size–we consider the scenario in which the product remains conserved between control and FCD Type I models.


$$v_{F\,C\,D}\cdot w_{F\,C\,D}=v_{C\,T\,L}\cdot w_{C\,T\,L}$$ (1)

$$\text{with}\,w_{C\,T\,L}\,<w_{F\,C\,D}\,\text{and}\,v_{C\,T\,L}>\,v_{F\,C\,D}$$

Under this constraint, the mean membrane potential *u* = is identical for both models (Gerstner et al., 2014) (Equation 8.26).


<u>∝v⁢w (2)

However, the variance of the membrane potential depends differently on synaptic parameters. The second moment scaled as (Gerstner et al., 2014) (Equation 8.27):


$$<△\,u^{2}>\propto w^{2}\,v$$ (3)

Under the conservation of mean input (Equation 1), this implies that conditions with larger individual EPSPs–such as those expected from hypertrophic spines in FCD Type I –exhibit increased membrane potential variance despite identical mean depolarization. As a result, the probability that membrane potential fluctuations cross spike threshold is increased in FCD Type I-like neurons. This conceptual mechanism (Equations 2, 3) is illustrated schematically in Figure 4C, where the membrane potential distribution for FCD Type I neurons (magenta dashed line) shows a heavier tail above threshold compared to control neurons (cyan solid line). This hypothesis provides a complementary perspective on how excitatory synaptic reorganization can increase firing probability without altering inhibition or average synaptic drive.

For computational modeling, all passive and active membrane parameters were constrained to remain consistent with previously reported electrophysiological measurements from human cortical pyramidal neurons (Cho et al., 2024; Eyal et al., 2016; Wilbers et al., 2023; Testa-Silva et al., 2022), ensuring that the model operated within a physiologically plausible regime. This strategy was adopted to prevent divergence between simulated and biological neurons and to enable mechanistic interpretations that are directly anchored to experimental observations.

A key feature of the model is the separation of spine-related effects into distinct but interacting mechanisms operating at different spatial scales. At the global level, reductions in spine density decreased total dendritic membrane surface area, thereby increasing somatic input resistance and amplifying voltage responses to synaptic input. This effect results in an additional increase in membrane potential variance in Equation 3, as illustrated by the magenta solid line in Figure 4C. However, the input-resistance increase observed in our model should be interpreted cautiously and may not generalize to all FCD subtypes. Previous studies reported reduced input resistance in cytomegalic and dysmorphic cells from cortical dysplasia tissue, cell types that are more characteristic of FCD Type II pathology (Cepeda et al., 2005, 2010). Thus, the passive mechanism proposed here–reduced spine density leading to decreased membrane area and increased input resistance–is most appropriately interpreted as a spine-mediated mechanism in pyramidal neurons, rather than as a general mechanism applicable to dysmorphic or cytomegalic neurons.

At the local level, changes in spine neck geometry selectively regulate electrical compartmentalization between the spine head and parent dendrite, redistributing synaptic voltage from the spine head toward the dendritic shaft. This aspect is particularly important because electrical compartmentalization within spines is thought to play a critical role in shaping local calcium dynamics and, in turn, synaptic plasticity (Bell et al., 2022). Extending this concept, a recent connectomics-based biophysical study proposed that spine neck resistance functions as a geometry-dependent control parameter that shapes the temporal dynamics of spinous postsynaptic potentials, high-frequency synaptic input tracking, and calcium signaling (Ofer et al., 2026). By systematically varying these features independently, we demonstrate that spine density and spine neck diameter exert separable and complementary influences on synaptic integration. Notably, spine neck diameter emerged as the dominant determinant of electrical coupling, whereas spine head size played a comparatively minor role, consistent with theoretical predictions of cable filtering in confined geometries (Araya et al., 2006).

These provide mechanistic insight into how spine alterations associated with FCD Type I. A prevailing view emphasizes reduced inhibition as a principal driver of hyperexcitability in epileptic cortex (Yang et al., 2021; Cho et al., 2024; Roper et al., 1999; Medici et al., 2016; Chen et al., 2025; Calcagnotto et al., 2005; Duma et al., 2024). In the present computational model, increased neuronal excitability was observed when alterations in excitatory spine architecture alone were considered. In particular, the structural reorganization of dendritic spines shifted the input-output relationship of individual neurons by lowering spike threshold and increasing gain in low-input regimes. These findings support the view that excitatory structural alterations may contribute to the hyperexcitable properties of FCD Type I tissue, although the present analysis was restricted to the specific spine parameters implemented in the model. Modeling these changes together with alterations in inhibitory signaling will be an important next step toward a more complete mechanistic understanding of epileptogenesis in FCD Type I. These results should be interpreted as simulation-based mechanistic comparisons rather than population-level statistical inference across biological specimens. Thus, the statistical analyses indicate the consistency of morphology-dependent effects within a defined computational cell model, but do not establish their magnitude or prevalence across biological populations. Within this limitation, the modeled spine alterations were sufficient to shift neuronal excitability in a direction consistent with hyperexcitability.

More broadly, this work highlights the importance of considering dendritic spine geometry as a distinct determinant of synaptic integration, separable from changes in synaptic strength. By explicitly dissociating geometric parameters from synaptic conductance in our simulations, we demonstrate that spine morphology alone can substantially reshape the flow of electrical signals across compartments. Disease-associated changes in spine geometry can therefore alter how synaptic inputs are integrated and transformed into action potentials, independent of concurrent changes in synaptic efficacy. By linking ultrastructural observations from human tissue directly to functional consequences at the cellular level, our study provides a framework for bridging structural pathology and electrophysiological dysfunction in epilepsy. Future extensions incorporating active dendritic conductances and network-level interactions will be important for determining how these spine-dependent mechanisms interact with inhibition and recurrent excitation to shape epileptiform activity in intact cortical circuits.

## Data Availability

All custom codes used for simulations, data analysis, and figure generation are available here: https://github.com/jawonGim/Simulation_SpineAlterationFocalCorticalDysplasia.git.
