# Supplementary material for: Microstructural spine alterations increase neuronal excitability in focal cortical dysplasia Type I
Source: Front Comput Neurosci. 2026 Aug 26;20:1862383. doi: 10.3389/fncom.2026.1862383 (PMC13553748; doi:10.3389/fncom.2026.1862383)
Supplement: Supplementary file 1 [file Data_Sheet_1.docx]

Supplementary Material

# 1 Supplementary Figures

**Figure S1. EPSP traces recorded during single-spine activation simulations.** (A) Schematic of recording sites: spine head (red), spine base (blue), and soma (black). EPSPs were recorded under five model configurations: (B) control, (C) altered spine density only, (D) altered spine neck diameter only, (E) altered spine head diameter only, and (F) all spine architectural parameters altered simultaneously. For each model configuration (B–F), the top row shows raw EPSP traces recorded at all three sites, and the bottom row shows an expanded view of the somatic EPSP. Light-colored traces represent individual simulation runs, and darker traces denote trial-averaged responses. 20 simulations were performed for each model configuration. Horizontal cyan dashed lines indicate the peak EPSP amplitude at the same recording site in the control configuration.

**Figure S2. Effect of spine neck length and diameter on EPSP compartmentalization.** (A–C) Heatmaps showing EPSP amplitude at the spine head (A), EPSP amplitude at the spine base (B), and the voltage difference between the spine head and base (ΔV_head–base_) (C) as functions of spine neck diameter and neck length. This analysis complements Figure 3 by varying spine neck length together with spine neck diameter. Each pixel in panels A–C represents the mean value from 30 independent simulations performed for the corresponding neck diameter–length combination. (D) Calculated spine neck axial resistance as a function of spine neck diameter and neck length. Neck resistance was calculated using the cytoplasmic resistivity (R_a_) listed in **Table S2**, as described in **Equation S3**.

# 2 Supplementary Tables

**Table S1.** **Morphological parameters of control and epileptogenic model cells (see Figure 1 and Methods Section 2.1-2.2).** For compartments without an explicitly specified nseg value, the number of segments was automatically determined based on the electrotonic length constant (λ) derived from the passive membrane properties as described in **Methods**. The soma and spine head were implemented as spherical compartments with the diameters provided in the table below.  Spine neck length was calculated by subtracting the spine head diameter from the total spine length. All spine morphology parameters were based on the mean values obtained from vEM-based quantitative analysis (G. H. Kim et al. 2025).

|  | | **Control** | **nseg** | **Epileptogenic** | **nseg** |
| --- | --- | --- | --- | --- | --- |
| **Soma** | **Diameter (μm)** | 17 | 3 | 17 | 3 |
| **Apical dendrite** | **Number of branches** | 14 | | | |
|  | **Length (μm)** | 381 | | | |
|  | **Diameter (μm)** | 1-4 (Tapered) | | | |
| **Basal dendrite** | **Number of dendrites** | 6 | | | |
|  | **Length (μm)** | 381 | | | |
|  | **Diameter (μm)** | 1 | | | |
| **Spine** | **Head diameter (μm)** | 0.551 | 1 | 0.718 | 1 |
|  | **Neck diameter (μm)** | 0.148 | 3 | 0.301 | 3 |
|  | **Total length (μm)** | 1.823 | | 1.696 | |
|  | **Total number** | 80x20 | | 30x20 | |

**Table S2. Biophysics and simulation parameters of control and epileptogenic model cells.** Parameters were initialized from literature-based values and refined to match action potential kinetics reported for FCD-derived electrophysiological recordings by Cho et al. 2024. See Methods Section 2.1.

|  | **Value** | **Unit** | **References** |
| --- | --- | --- | --- |
| **R_a_ (Cytoplasmic resistivity)** | 203.23 | Ω·cm | Eyal et al. 2016 |
| **R_m_ (Membrane resistivity)** | 38,907 | Ω·cm² | Eyal et al. 2016 |
| **C_m_ (Membrane capacitance)** | 1 | µF/cm² | Testa-Silva et al. 2022 |
| **e_pas_ (Passive equilibrium)** | -70 | mV | Wilbers et al. 2023 |
| **Na+ channel conductance** | 11,000 | pS/μm² | Obi-Nagata et al. 2023 |
| **K+ channel conductance** | 2,100 | pS/μm² | Obi-Nagata et al. 2023 |
| **Temperature** | 37 | ℃ | Eyal et al. 2016, Obi-Nagata et al. 2023 |
| **AMPAR weights _control** | 0.31 | nS | Cho et al. 2024, Obi-Nagata et al. 2023, Testa-Silva et al. 2022 |
| **AMPAR weights _epileptogenic** | 0.82 | nS | Cho et al. 2024, Obi-Nagata et al. 2023, Testa-Silva et al. 2022 |
| **NMDAR weights _control** | 0.63 | nS | Cho et al. 2024, Obi-Nagata et al. 2023, Testa-silva 2022 |
| **NMDAR weights _epileptogenic** | 1.62 | nS | Cho et al. 2024, Obi-Nagata et al. 2023, Testa-Silva et al. 2022 |

# 3 Supplementary Equations

**Equation S1. Calculation of dendritic shaft surface area.** Dendritic sections were modeled as cylinders with constant diameter. The dendritic shaft surface area was calculated from the lateral surface area of a cylinder, with the attachment areas occupied by spine necks subtracted to avoid double-counting the spine–dendrite interface.

$$dendritic shaft surface area=\left( \pi\times dendrite diameter \times dendrite length \right)-\left\{ \pi\left( \frac{spine neck diameter}{2} \right)^{2}\times number of spines \right\}$$

**Equation S2. Calculation of spine surface area.** Dendritic spines were modeled as a spherical head connected to a cylindrical neck. Spine surface area was calculated as the sum of the spherical head surface area and the lateral surface area of the cylindrical neck, with the neck attachment area on the spine head subtracted.

$$spine surface area=\left\{ 4\pi\left( \frac{spine head diameter}{2} \right)^{2}-\pi\left( \frac{spine neck diameter}{2} \right)^{2}+\left( \pi\times spine neck width\times spine neck length \right) \right\}\times number of spines$$

**Equation S3. Calculation of spine neck axial resistance used for Figure 2C, S2.** Spine neck resistance was calculated by treating the spine neck as a cylindrical cable. Cytoplasmic resistivity (Rₐ) was taken from **Table S2**. In the equation below, R_a_ is in Ω·cm, and spine neck length and diameter are in μm; the resulting resistance is expressed in MΩ.

$$Neck Resistance(M\Omega)=\frac{R_{a}\times neck length\times{10}^{-4}}{\left\{ \pi\times\left( \frac{neck diameter\times{10}^{-4}}{2} \right)^{2} \right\}}\times{10}^{-6}$$
